# Supplementary figures and images for: “SDM:HOSP”- a generic model for hospital-based implementation of shared decision making
Source: PLoS One. 2023 Jan 24;18(1):e0280547. doi: 10.1371/journal.pone.0280547 (PMC9873173; doi:10.1371/journal.pone.0280547)

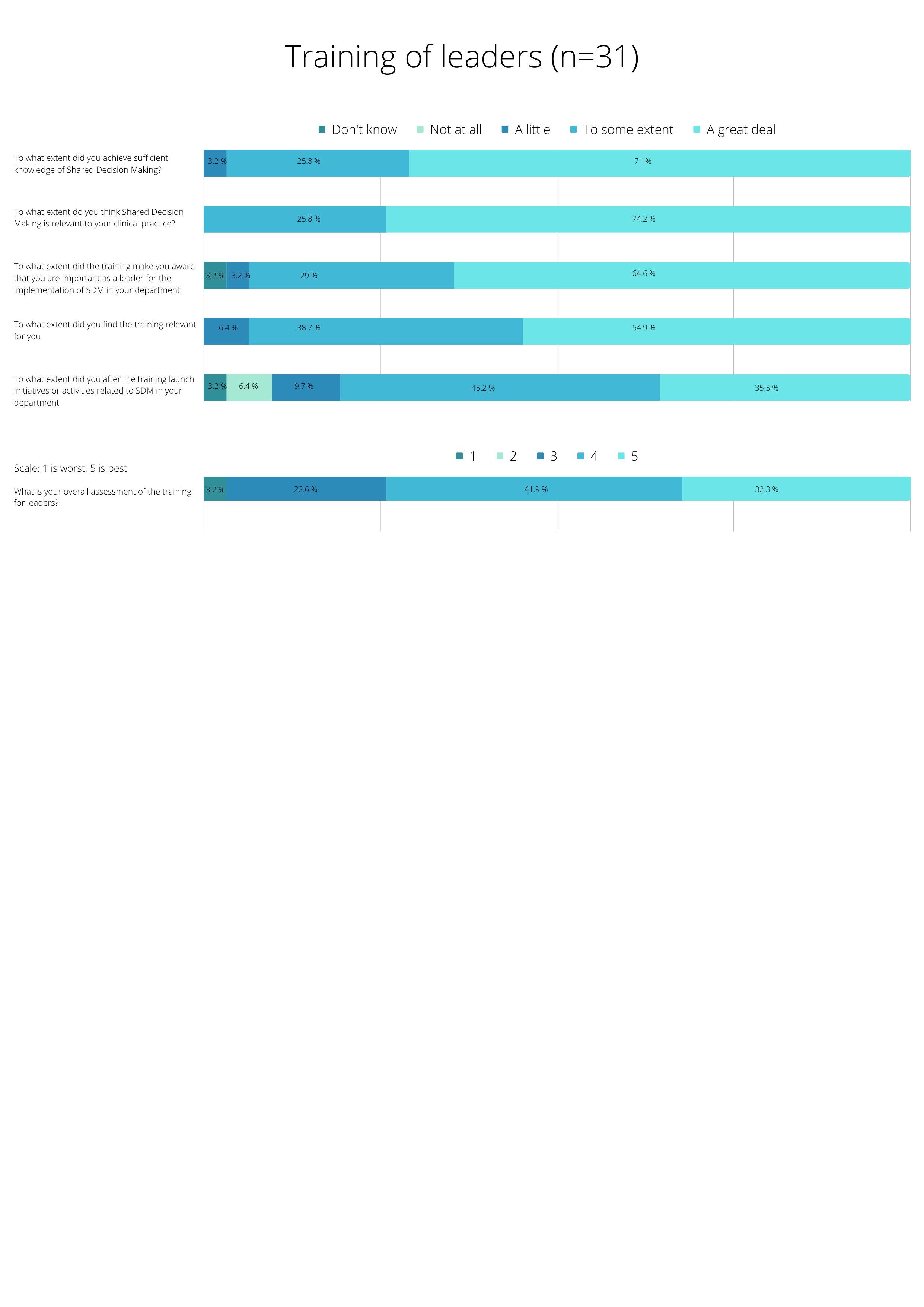

Supplement: S1 Fig — (TIFF) [file pone.0280547.s002.tiff]

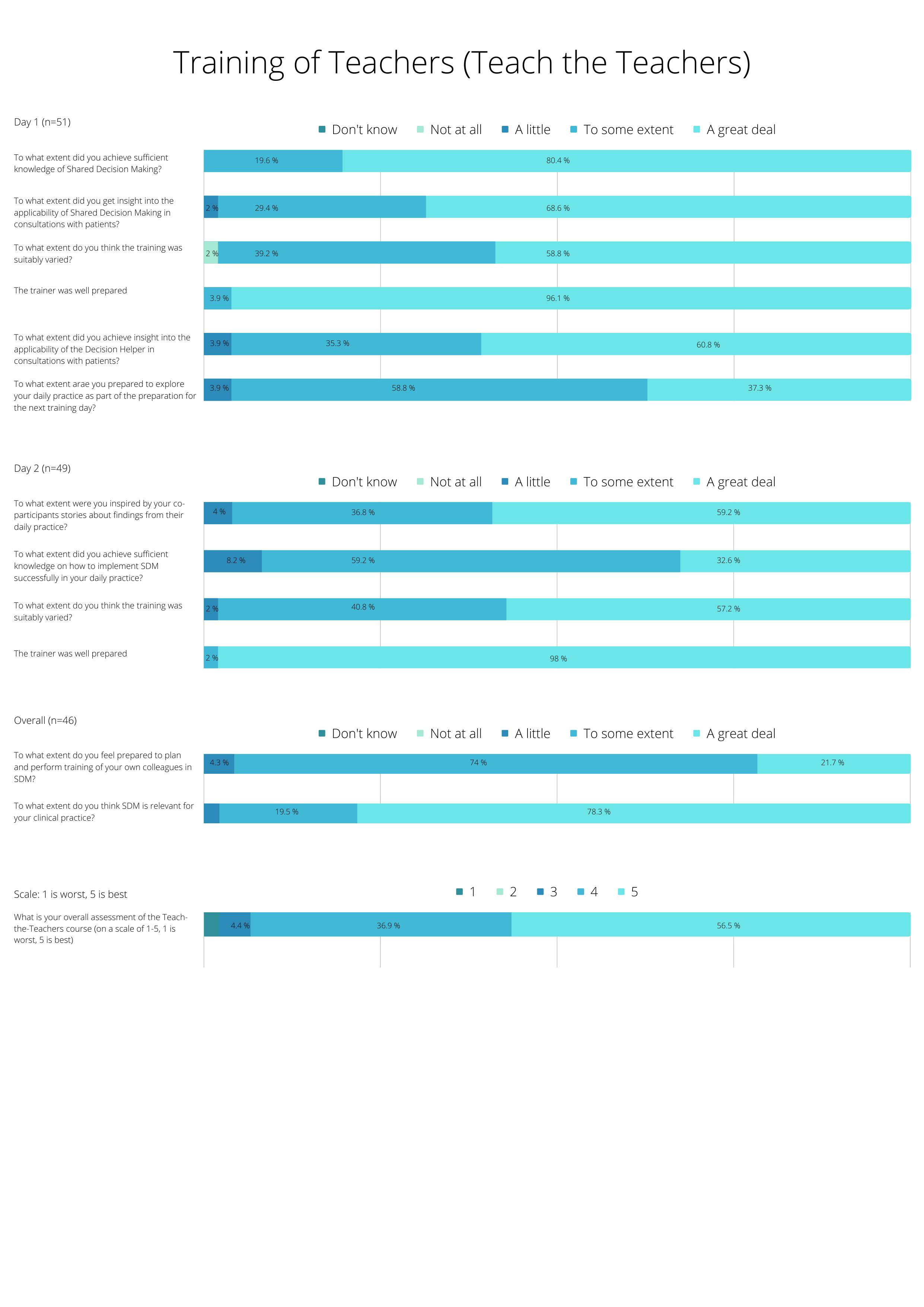

Supplement: S2 Fig — (TIFF) [file pone.0280547.s003.tiff]

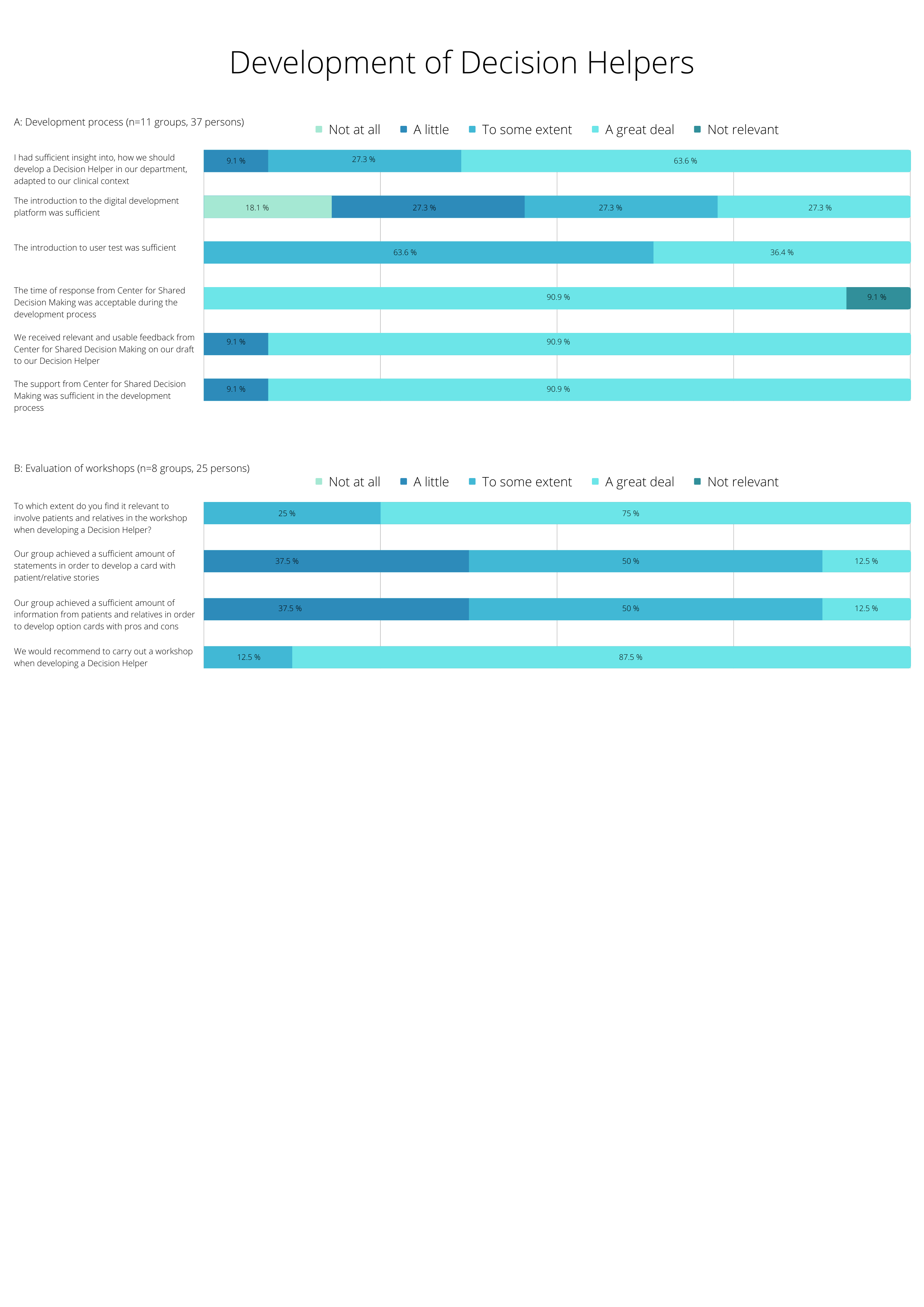

Supplement: S3 Fig — (TIFF) [file pone.0280547.s004.tiff]

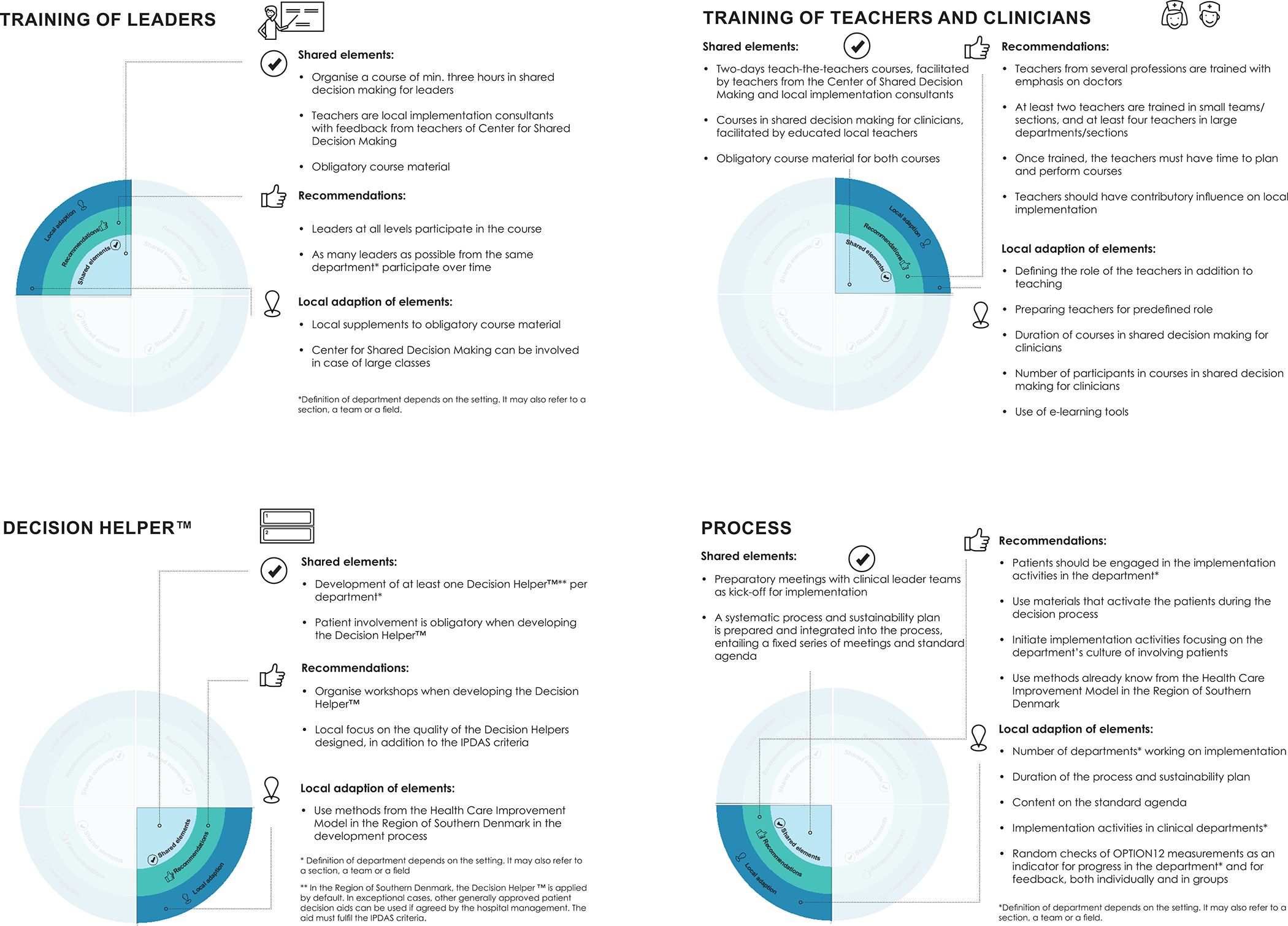

Supplement: S4 Fig — A-D. Elaborated themes within SDM:HOSP. (TIFF) [file pone.0280547.s005.tiff]

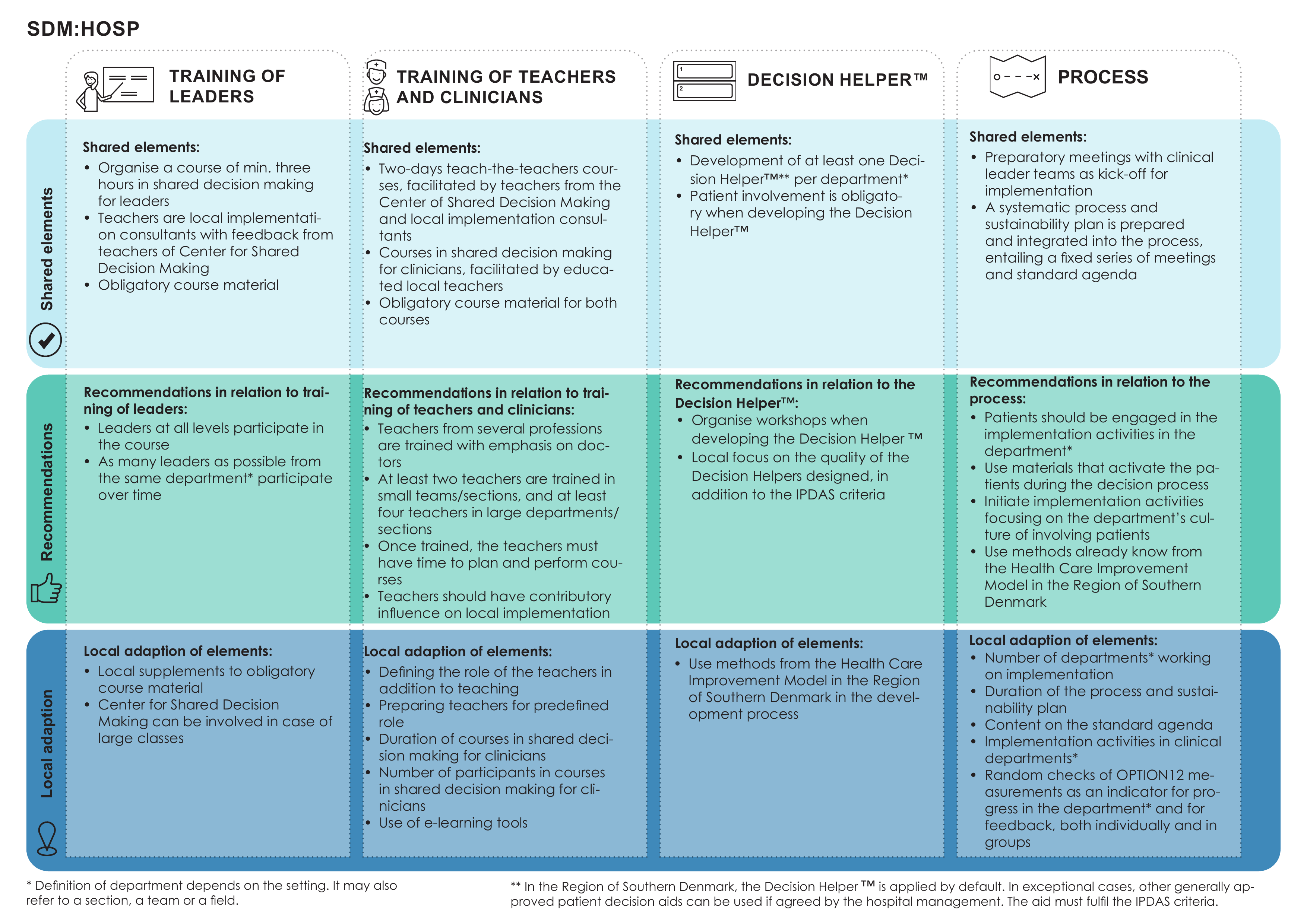

Supplement: S5 Fig — (TIFF) [file pone.0280547.s006.tiff]
