# Supplementary material for: “SDM:HOSP”- a generic model for hospital-based implementation of shared decision making
Source: PLoS One. 2023 Jan 24;18(1):e0280547. doi: 10.1371/journal.pone.0280547 (PMC9873173; doi:10.1371/journal.pone.0280547)
Supplement: S1 Table — (DOCX) [file pone.0280547.s007.docx]

| **Training of Leaders (n=31)** | **Don’t**  **know** | **Not at all** | | | **A little** | | | **To some extent** | | | **A great deal** | | |
| --- | --- | --- | --- | --- | --- | --- | --- | --- | --- | --- | --- | --- | --- |
|  |  |  | | |  | | |  | | |  | | |
| To what extent did you achieve sufficient knowledge of Shared Decision Making | 0 |  | 0 |  |  | 1 |  |  | 8 |  |  | 22 |  |
| To what extent do you think Shared Decision Making is relevant to your clinical practice | 0 |  | 0 |  |  | 0 |  |  | 8 |  |  | 23 |  |
| To what extent did the training make you aware that you are important as a leader for the implementation of SDM in your department | 1 |  | 0 |  |  | 1 |  |  | 9 |  |  | 20 |  |
| To what extent did you find the training relevant for you | 0 |  | 0 |  |  | 2 |  |  | 12 |  |  | 17 |  |
| To what extent did you after the training launch initiatives or activities related to SDM in your department | 1 |  | 2 |  |  | 3 |  |  | 14 |  |  | 11 |  |
|  |  |  |  |  |  |  |  |  |  |  |  |  |  |
| Scale: 1 is worst, 5 is best | **1** |  | **2** |  |  | **3** |  |  | **4** |  |  | **5** |  |
| What is your overall assessment of the training for leaders | 1 |  | 0 |  |  | 7 |  |  | 13 |  |  | 10 |  |
