# Supplementary material for: “SDM:HOSP”- a generic model for hospital-based implementation of shared decision making
Source: PLoS One. 2023 Jan 24;18(1):e0280547. doi: 10.1371/journal.pone.0280547 (PMC9873173; doi:10.1371/journal.pone.0280547)
Supplement: S2 Table — (DOCX) [file pone.0280547.s008.docx]

| **Training of Teachers (Teach the Teachers)** | Don’t know | Not at all | | | A little | | | To some extent | | | A great deal | | |
| --- | --- | --- | --- | --- | --- | --- | --- | --- | --- | --- | --- | --- | --- |
| **Day 1 (n=51)** |  |  | | |  | | |  | | |  | | |
| To what extent did you achieve sufficient knowledge of Shared Decision Making | 0 |  | 0 |  |  | 0 |  |  | 10 |  |  | 41 |  |
| To what extent did you get insight into the applicability of Shared Decision Making in consultations with patients | 0 |  | 0 |  |  | 1 |  |  | 15 |  |  | 35 |  |
| To what extent do you think the training was suitably varied | 0 |  | 1 |  |  | 0 |  |  | 20 |  |  | 30 |  |
| The trainer was well prepared | 0 |  | 0 |  |  | 0 |  |  | 2 |  |  | 49 |  |
| To what extent did you achieve insight into the applicability of the Decision Helper in consultations with patients | 0 |  | 0 |  |  | 2 |  |  | 18 |  |  | 31 |  |
| To what extent are you prepared to explore your daily practice as part of the preparation for the next training day | 0 |  | 0 |  |  | 2 |  |  | 30 |  |  | 19 |  |
| **Day 2 (n=49)** |  |  |  |  |  |  |  |  |  |  |  |  |  |
| To what extent were you inspired by your co-participants stories about findings from their daily practice | 0 |  | 0 |  |  | 2 |  |  | 18 |  |  | 29 |  |
| To what extent did you achieve sufficient knowledge on how to implement SDM successfully in your daily practice | 0 |  | 0 |  |  | 4 |  |  | 29 |  |  | 16 |  |
| To what extent do you think the training was suitably varied | 0 |  | 0 |  |  | 1 |  |  | 20 |  |  | 28 |  |
| The trainer was well prepared | 0 |  | 0 |  |  | 0 |  |  | 1 |  |  | 48 |  |
| **Overall (n=46)** |  |  |  |  |  |  |  |  |  |  |  |  |  |
| To what extent do you feel prepared to plan and perform training of your own colleagues in SDM | 0 |  | 0 |  |  | 2 |  |  | 34 |  |  | 10 |  |
| To what extent do you think SDM is relevant for your clinical practice | 0 |  | 0 |  |  | 1 |  |  | 9 |  |  | 36 |  |
|  |  |  |  |  |  |  |  |  |  |  |  |  |  |
| Scale: 1 is worst, 5 is best | **1** |  | **2** |  |  | **3** |  |  | **4** |  |  | **5** |  |
| What is your overall assessment of the Teach-the-Teachers course (on a scale of 1-5, 1 is worst, 5 is best) | 1 |  | 0 |  |  | 2 |  |  | 17 |  |  | 26 |  |
