# Supplementary material for: “SDM:HOSP”- a generic model for hospital-based implementation of shared decision making
Source: PLoS One. 2023 Jan 24;18(1):e0280547. doi: 10.1371/journal.pone.0280547 (PMC9873173; doi:10.1371/journal.pone.0280547)
Supplement: S3 Table — (DOCX) [file pone.0280547.s009.docx]

| **Development of Decision Helpers** | | | | | | | | | | | | | | | |
| --- | --- | --- | --- | --- | --- | --- | --- | --- | --- | --- | --- | --- | --- | --- | --- |
|  | Not at all | | | A little | | | To some extent | | | A great deal | | | Not relevant | | |
| **A:** **Development process (n = 11 groups, 37 persons)** | | | | | | | | | | | | | | | |
| I had sufficient insight into, how we should develop a Decision Helper in our department, adapted to our clinical context |  | 0 |  |  | 1 |  |  | 3 |  |  | 7 |  |  | 0 |  |
| The introduction to the digital development platform was sufficient |  | 2 |  |  | 3 |  |  | 3 |  |  | 3 |  |  | 0 |  |
| The introduction to user test was sufficient |  | 0 |  |  | 0 |  |  | 7 |  |  | 4 |  |  | 0 |  |
| The time of response from Center for Shared Decision Making was acceptable during the development process |  | 0 |  |  | 0 |  |  | 0 |  |  | 10 |  |  | 1 |  |
| We received relevant and usable feedback from Center for Shared Decision Making on our draft to our Decision Helper |  | 0 |  |  | 1 |  |  | 0 |  |  | 10 |  |  | 0 |  |
| The support from Center for Shared Decision Making was sufficient in the development process |  | 0 |  |  | 1 |  |  | 0 |  |  | 10 |  |  | 0 |  |
| **B: Evaluation of workshops (n = 8 groups, 25 persons)** | | | | | | | | | | | | | | | |
| To which extent do you find it relevant to involve patients and relatives in the workshop when developing a Decision Helper |  | 0 |  |  | 0 |  |  | 2 |  |  | 6 |  |  | 0 |  |
| Our group achieved a sufficient amount of statements in order to develop a card with patient/relative stories |  | 0 |  |  | 3 |  |  | 4 |  |  | 1 |  |  | 0 |  |
| Our group achieved a sufficient amount of information from patients and relatives in order to develop option cards with pros and cons |  | 0 |  |  | 3 |  |  | 4 |  |  | 1 |  |  | 0 |  |
| We would recommend to carry out a workshop when developing a Decision Helper |  | 0 |  |  | 0 |  |  | 1 |  |  | 7 |  |  | 0 |  |
