# Supplementary material for: “SDM:HOSP”- a generic model for hospital-based implementation of shared decision making
Source: PLoS One. 2023 Jan 24;18(1):e0280547. doi: 10.1371/journal.pone.0280547 (PMC9873173; doi:10.1371/journal.pone.0280547)
Supplement: S4 Table — (DOCX) [file pone.0280547.s010.docx]

| **Overall evaluation** | **Don’t know** | **Not at all** | | | **A little** | | | **To some extent** | | | **Quite a bit** | | | **A great deal** | | |
| --- | --- | --- | --- | --- | --- | --- | --- | --- | --- | --- | --- | --- | --- | --- | --- | --- |
|  |  |  | | |  | | |  | | |  | | |  | | |
| To what extent did you use a process plan during your implementation (n=87) | 1 |  | 1 |  |  | 28 |  |  | 4 |  |  | 50 |  |  | 3 |  |
| How useful do you think a pre-meeting with department managers is (n=38) | 2 |  | 1 |  |  | 1 |  |  | 0 |  |  | 11 |  |  | 23 |  |
| A fixed series of meetings is relevant during the implementation process (n=38) | 5 |  | 0 |  |  | 1 |  |  | 4 |  |  | 14 |  |  | 14 |  |
| Standard agendas to all fixed meetings are relevant to the implementation process (n=38) | 2 |  | 0 |  |  | 2 |  |  | 6 |  |  | 13 |  |  | 15 |  |
| To what extent do you think the course “Shared Decision Making for Managers” is useful in the implementation process (n=21) | 0 |  | 0 |  |  | 1 |  |  | 4 |  |  | 5 |  |  | 11 |  |
| To what extent do you think the course “Shared Decision Making - Teach-the-Teachers” is useful in the implementation process (n=27) | 2 |  | 0 |  |  | 0 |  |  | 5 |  |  | 7 |  |  | 13 |  |
| To what extent do you think the course “Shared Decision Making for clinicians” is useful in your department (n=58) | 1 |  | 0 |  |  | 7 |  |  | 6 |  |  | 21 |  |  | 23 |  |
| To what extent do you think developing and implementing a Decision Helper is useful for the implementation process (n=34) | 1 |  | 0 |  |  | 3 |  |  | 9 |  |  | 11 |  |  | 10 |  |
| To what extent did you find support from Center for Shared Decision Making useful in your development of a Decision Helper or implementation process (n=25) | 2 |  | 0 |  |  | 0 |  |  | 3 |  |  | 9 |  |  | 11 |  |
| To what extent was cooperating with your local consultant useful (n=62) | 3 |  | 0 |  |  | 2 |  |  | 7 |  |  | 26 |  |  | 24 |  |
| To what extent did implementing Shared Decision Making affect your department positively, at this time point (77) | 10 |  | 5 |  |  | 8 |  |  | 33 |  |  | 18 |  |  | 3 |  |
| To what extent did you involve patients and/or relatives in your implementation process (n=77) | 4 |  | 10 |  |  | 0 |  |  | 32 |  |  | 31 |  |  | 0 |  |
| To what extent did your attitude towards involving patients and/or relatives change, after participating in a course or development of a Decision Helper (n=58) | 16 |  | 1 |  |  | 10 |  |  | 16 |  |  | 13 |  |  | 2 |  |
| To what extent do you agree that Shared Decision Making is essential to patients at your hospital unit or department (n=87) | 1 |  | 3 |  |  | 5 |  |  | 14 |  |  | 26 |  |  | 38 |  |
| To what extent do you agree that Shared Decision Making is essential to relatives at your hospital unit or department (n=87) | 1 |  | 4 |  |  | 16 |  |  | 16 |  |  | 22 |  |  | 28 |  |
| To what extent do you agree that Shared Decision Making is essential to health care professionals at your hospital unit or department (n=87) | 1 |  | 2 |  |  | 6 |  |  | 20 |  |  | 26 |  |  | 32 |  |
